# Study of mesoporous CdS-quantum-dot-sensitized TiO_2_ films by using X-ray photoelectron spectroscopy and AFM

**DOI:** 10.3762/bjnano.5.6

**Published:** 2014-01-20

**Authors:** Mohamed Nawfal Ghazzal, Robert Wojcieszak, Gijo Raj, Eric M Gaigneaux

**Affiliations:** 1 Institute of Condensed Matter and Nanoscience – Molecules, Solids and Reactivity (IMCN/MOST), Université Catholique de Louvain, Croix du Sud 2/17, 1348 Louvain-La-Neuve, Belgium; 2 Université de Namur, Technology Transfert Office, rue de Bruxelles 61 - 5000 Namur, Belgique; 3 Institute of Chemistry, University of Sao Paulo, USP, São Paulo, 05508-000, SP, Brazil

**Keywords:** AFM, CdS, heterojunction, particle size, quantum dots, TiO_2_, XPS

## Abstract

CdS quantum dots were grown on mesoporous TiO_2_ films by successive ionic layer adsorption and reaction processes in order to obtain CdS particles of various sizes. AFM analysis shows that the growth of the CdS particles is a two-step process. The first step is the formation of new crystallites at each deposition cycle. In the next step the pre-deposited crystallites grow to form larger aggregates. Special attention is paid to the estimation of the CdS particle size by X-ray photoelectron spectroscopy (XPS). Among the classical methods of characterization the XPS model is described in detail. In order to make an attempt to validate the XPS model, the results are compared to those obtained from AFM analysis and to the evolution of the band gap energy of the CdS nanoparticles as obtained by UV–vis spectroscopy. The results showed that XPS technique is a powerful tool in the estimation of the CdS particle size. In conjunction with these results, a very good correlation has been found between the number of deposition cycles and the particle size.

## Introduction

To sensitize the photocatalyst TiO_2_ with cadmium sulfide quantum dots (QDs-CdS) is a well-established concept that is of great relevance in different applications. The most popular of these applications are photosensitized solar cells with high quantum yields [1–4] and the photocatalytic degradation of pollutants [5–6]. CdS, currently used as an efficient visible-light sensitizer, is a semiconductor that possesses a small band gap (2.4 eV) and suitable potential energies. The electron transfer between QDs-CdS and TiO_2_ is due to the different energy levels of the different conduction and valence bands [7]. This transfer takes place if an exciton is generated by the absorption of an incident photon. If the conduction band energy of CdS is higher than that of TiO_2_ the electron can be ejected [6].

Several studies reported the strong dependence of the photovoltaic conversion yield and photocatalytic efficiency on the particle size TiO_2_ sensitized with QDs-CdS [3,8]. Varying the size of the CdS particles allows for a tuning of the band gap energy of the QDs-CdS in order to reach the required value to sensitize TiO_2_. The suitable positions of the potential energies allow for an easy transfer of the exciton between the semiconductors. Not only does that help to optimize the charge separation by reducing the recombination of charges, it also allows for an extension of the photoresponse of the photocatalyst in the visible range. In general, the conventional methods that are used to estimate the average particle size of QDs-CdS are transmission electron microscopy (TEM) [4,8] or X-ray diffraction (XRD) [6], and UV–vis [9] spectroscopy to some extent. The main difficulty when working with very small particles (below 10 nm) is the determination of their exact size [9]. Because of the different morphology, the heterogeneous distribution on the surface and also the support effect some techniques are limited in their use for determination of size. While XRD is restricted by several factors such as the weight fraction or the crystallinity of the sample, TEM is limited by contrast effects between active phase and support [10]. Moreover, in order to get a correct size distribution several images of the same sample at different sites need to be analyzed and a huge number (about 1000) of particles need to be counted [9]. In the case of spectroscopy techniques such as UV–vis spectroscopy combined with effective mass approximation the values of particle size are usually strongly overestimated [11]. However, this technique could be useful in explaining the dependence of the band gap on quantum size effects [9,11–13].

In this study, X-ray photoelectron spectroscopy (XPS) was used for the first time, to the best of our knowledge, to estimate the particle size of QDs-CdS grown on a mesoporous TiO_2_ film. The successive ionic layer adsorption and reaction processes, which are defined as deposition cycles, have been applied to get QDs-CdS with variable particle sizes. For the purpose of validation, the results of the particle sizes obtained from XPS are compared to the results obtained from AFM analysis, and to the evolution of the band gap energy of CdS nanoparticles.

## Experimental

### TiO_2_ Film preparation

Mesoporous TiO_2_ films were prepared following the procedure reported elsewhere [14]. An adequate amount of titanium(IV) tetraethoxide (TEOT, Ti(OC_2_H_5_)_4_, 95% Aldrich) was dissolved under vigourous stirring (20 min) in concentrated hydrochloric acid (37%) at room temperature. In parallel, the hybrid solution was obtained by the addition of dissolved polyethylene glycol hexadecyl ether (denoted Brij 56, C_16_H_33_(OCH_2_CH_2_)*_n_*OH, *n* ≈ 10, Aldrich) into 1-butanol (BuOH, >99.4%, Alfa Aesar). The final molar ratio of the solution was TEOT/HCl/1-butanol/Brij 56 1:2–4:9:0.05. The solutions were subsequently aged under magnetic stirring at room temperature for 3 h before the films were spin-coated onto soda lime glass (SLG). Prior to use, the substrates were cleaned by ultrasonication (detergent, distilled water, acetone, ethanol, for 15 min in each medium) to remove hydrophobic contaminants at the surface and then air-dried at 150 °C. After the SLG was spin-coated with the hybrid sol with a spin speed of 2000 rpm, the coating was aged at room temperature for 12 h under atmospheric conditions. The xerogel was finally dried at increasing temperatures (6 h at 70 °C, 3 h at 150 °C and 2 h at 200 °C). Mesoporous titania films (degradation of the template agent and inorganic network consolidation) were then obtained by calcination in air at 400 °C over 2 h with a rising step of 1 °C min^−1^.

#### Preparation of QDs-CdS-sensitized TiO_2_

QDs-CdS were prepared following the procedure previously described by Besson et al. [9]. Briefly, the titania films were dipped for 1 min into a saturated nitrate solution of Cd^2+^ and washed with water for several times in order to eliminate excess reactive species. The deposition of Cd^2+^ was performed under controlled pH (≈10), which was adjusted by adding NaOH solution at 1 M. The chemical process enables a homogeneous adsorption of cationic species in Ti−O^−^ walls [9]. The resulting film was put in a sealed quartz tube under Argon flux, and gaseous H_2_S was injected slowly until *P*_H2S_ = *P*_atm_. These two steps (impregnation and precipitation) were repeated until the film was saturated. From here on, this procedure will be referred to as one coating; particles of different sizes were obtained by repeating the cycle of the coating procedure. The film impregnated with Cd^2+^ was colorless. After the first H_2_S treatment, the film became a light yellow color, and the color intensity increased during the following cycles.

#### Films characterization

TEM analysis was performed by using a LEO922 electron microscope operating at 200 keV. The film was scratched off from the substrate, dispersed in ethanol and subsequently deposited on copper grids coated with a porous carbon film. The solvent was evaporated in air prior to the analysis of the samples. AFM experiments were performed analogously to [15] by using a Nanoscope V multimode AFM (NanoSurfaces Business, Bruker Corporation, Santa Barbara, CA) in tapping mode (TM-AFM). Etched Si tapping mode cantilevers (TESP type, Bruker AFM probes), with a nominal curvature radius of 8 nm were used for imaging under ambient conditions (23 °C and 56% relative humidity). Samples were glued onto a magnetic stainless steel disc by using double-face adhesive tape and mounted on the "J" type piezoelectric scanner. The tapping engage set point was set to 1 in order to apply a minimal force to prevent sample deformation during imaging. The images where recorded at a scan rate of 0.5 Hz. The captured raw images were analyzed by using the Nanoscope scan analysis software (Bruker) and flattened to the 0th order to remove any underlying surface curvature. Similarly as described in [16], diffuse reflectance spectra of CdS/titania films were recorded by using an UV–vis spectrophotometer (Carry 5), which was equipped with an integrating sphere. The baseline was set by BaSO_4_ in the diffuse reflectance mode. The spectra were recorded at room temperature in the spectral range of interest 200–550 nm. XPS analysis was performed on Kratos Axis-ultra spectrometer. Similarly as described in [10], the analysis chamber was operated under ultrahigh vacuum conditions with an approximate pressure of 5 × 10^−7^ Pa and the sample was irradiated with a monochromatic Al Kα (1486.6 eV) radiation (10 kV; 22 mA). Charge stabilization was achieved by using an electron flood gun adjusted at 8 eV and placing a nickel grid 3 mm above the sample. Pass energy for the analyzer was set to 160 eV for wide scan. The analyzed area was approximately 1.4 mm^2^ and the pass energy was set to 50 eV for recording high resolution peaks. In these conditions, the full width at half maximum (FWHM) of the Au 4f_7/2_ peak of a clean gold standard sample was about 1.1 eV. The surface atomic concentrations were calculated by correcting the intensities with theoretical sensitivity factors based on Scofield cross-sections [6] and the mean free path varying according to the 0.7th power of the photoelectron kinetic energy. Peak deconvolution was performed by using curves with a 70% Gaussian type and a 30% Lorentzian type, and a Shirley non-linear sigmoid-type baseline. The following peaks were used for the quantitative analysis: O 1s, C 1s, Ti 2p and Cd 3d, Cd 4s and Cd 3s. The Cl 2p, S 2p and N 1s peaks were also monitored and C 1s to check for charge stability as a function of time. CdS (from Fluka, 99.9% analytical grade) was used as the reference material for the study of the prepared materials. For Kratos measurements, (i) sample powders were pressed into small stainless steel troughs mounted on a multi specimen holder; (ii) the C−(C,H) component of the C 1s peak of adventitious carbon was fixed to 284.8 eV to set the binding energy scale; (iii) the data were analyzed using the CasaXPS software (CasaSoftware Ltd, UK).

## Results and Discussion

### AFM and TEM images

**Figure 1:**
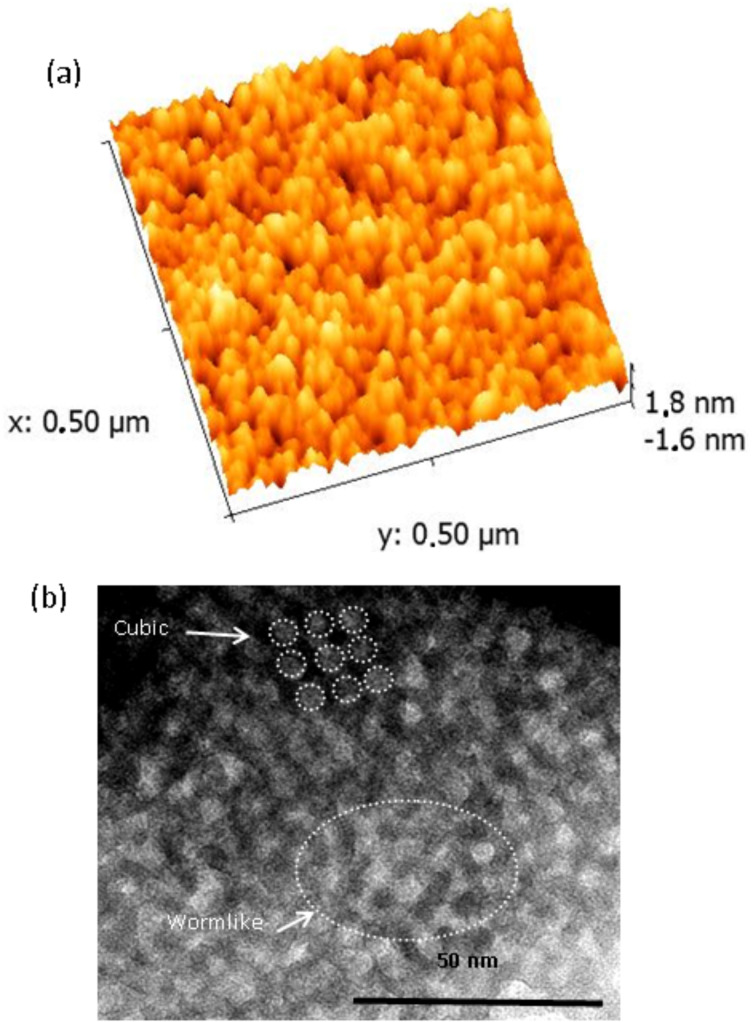
(a) AFM image of the mesoporous TiO_2_ film and (b) TEM image showing the ordered–desordered regions of the mesoporous TiO_2_ film.

Figure 1a shows the AFM height image of the TiO_2_ film with a root mean square (rms) roughness of less than 1 nm. The pore openings are relatively well distributed on the surface with an average size of ca. 6 nm. Figure 1b shows the TEM micrographs of the TiO_2_ films obtained by using Brij 56 as template agent. The film shows a homogeneous mesoporous size partially with ordered–disordered regions. The pore size is fairly comparable to that observed in AFM.

The mesoporous TiO_2_ films were exposed to Cd^2+^ and S^2−^ ions by successive immersions in a solution of Cd(NO_3_)_2_, H_2_S and water. In order to assess the deposition/growth process, we followed the CdS deposition on mesoporous TiO_2_ film by monitoring the absorption spectra, AFM images and XPS (as a new technique to efficiently evaluate the particle size of CdS) at different stages. The successive layers of CdS were deposited onto the TiO_2_ film for up to 15 deposition cycles (1, 3, 5, 7, 9 and 15). The deposition time was fixed at 60 s, which was reported as the necessary duration of the nucleation stage [17]. The formation of a Cd(OH)_2_ thin layer occurs during this stage and the CdS layer grows on it after exposure to H_2_S. Upon completion of each cycle, CdS nanoparticles are deposited onto the TiO_2_ surface as a layer [3] or localized into the mesoporous structure [9].

**Figure 2:**
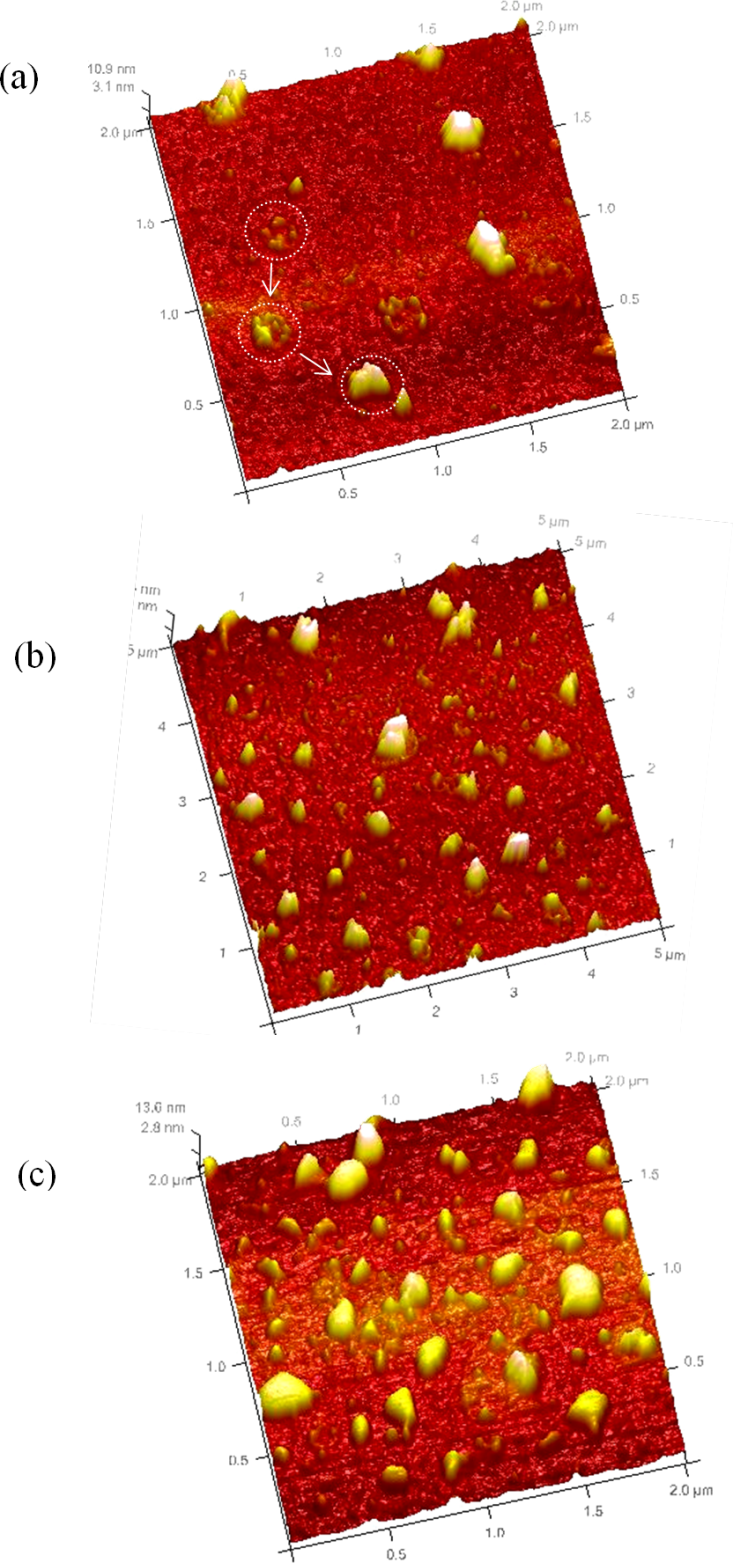
AFM images showing size evolution of CdS particles grown on mesoporous TiO_2_ with different number of deposition cycles (a) 5×CdS/TiO_2_, (b) 7×CdS/TiO_2_ and (c) 15×CdS/TiO_2_.

AFM analysis performed after 1 to 3 deposition cycles (result not shown) did not show the presence of CdS nanoparticles at the surface of the titanium dioxide films. This result contradicts that obtained by using XPS surface analysis performed on the films, which confirmed the presence of CdS nanoparticles (Table 1, see below). The formation of CdS inside the films pores could explain the discrepancy. Consequently, until up to 3 deposition cycles, CdS nanoparticles probably grow inside the pores of the films and no nanoparticles are observed on the surface. After 3 deposition cycles, AFM images show the presence of CdS nanoparticles on the surface of TiO_2_ films. The size of the particles increased with the number of the deposition cycles (5, 7 and 15 deposition cycles). Two kinds of crystals were observed for five deposition cycles (5×CdS/TiO_2_) (Figure 2a): separately dispersed CdS nanocrystal behind the very small CdS particles regrouped in aggregates. The formation of the aggregates could result from the accumulation of separated CdS crystals. The size of isolated crystals was smaller than 5 nm as measured from AFM cross-section. With the increase of CdS deposition cycles, the average particle size increased to 8 nm for 7×CdS/TiO_2_ (Figure 2b), and 10 nm for 15×CdS/TiO_2_ (Figure 2c). Of note is that despite the presence of few isolated crystallites (5 nm high), the lateral size of the crystals after 15 deposition cycles was remarkably larger than after 7 deposition cycles. This shows that increasing the number of deposition cycles leads to the growth of CdS nanocrytals in two forms; 1) the formation of new crystallites at each depositing cycle, and 2) the growth of pre-deposited crystallites into large aggregates.

**Figure 3:**
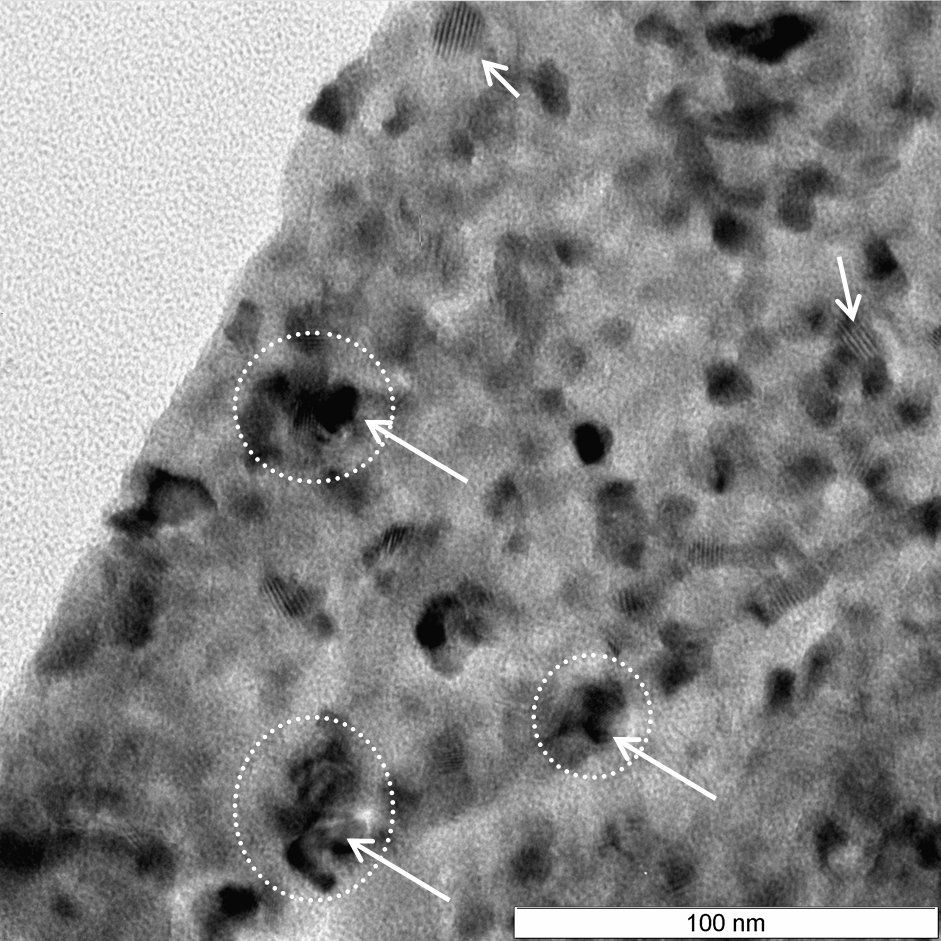
TEM image of the for 15×CdS/TiO_2_ sample.

TEM analysis was performed for the 15×CdS/TiO_2_ sample (Figure 3). It was found that the majority of CdS nanoparticles have a nearly spherical shape with an average particle size of about 10 nm. The TEM study showed the presence of aggregates as a result of spherical particles accumulation, which confirmed our previous hypothesis. The aggregates remain separated from each other, and grow to a diameter of approx. 20 nm. Our data indicate that the growth of the particles inside the pores and the formation of aggregates make the estimation of the average particle size of the CdS nanoparticles by AFM very challenging and result in overestimated values.

### UV–vis diffuse reflectance spectroscopy

**Figure 4:**
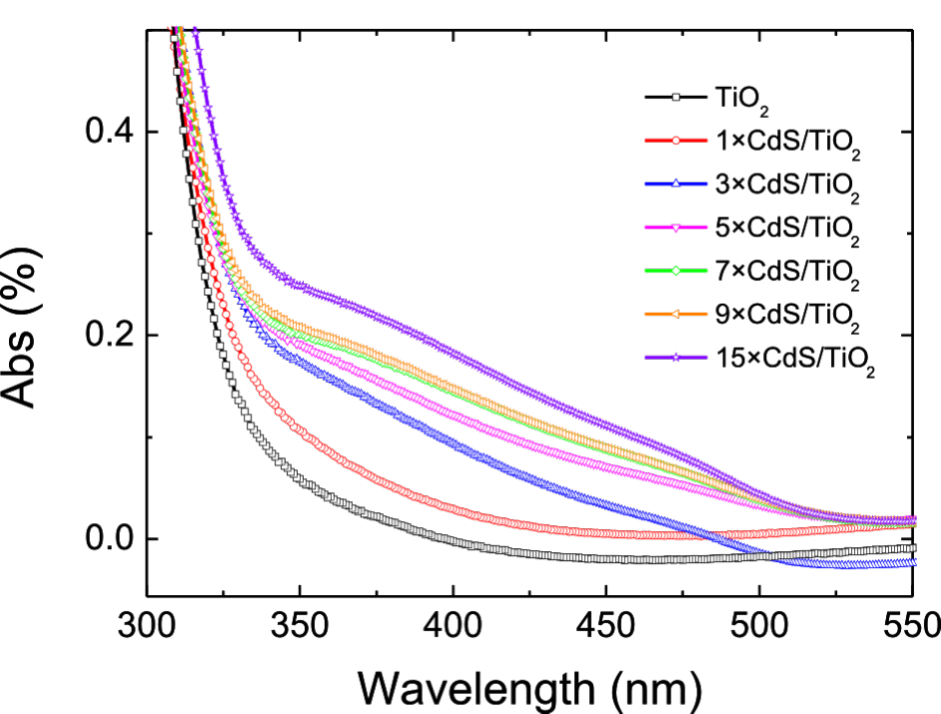
Absorption spectra of the CdS-sensitized titanium dioxide films after different numbers of deposition cycles.

The absorption spectra recorded for various increasing deposition cycles of CdS quantum dots are shown in Figure 4. The TiO_2_ film absorbs only in the UV range (λ < 375 nm) whereas the absorption edge is shifted to red with successive CdS deposition cycles. The CdS-sensitized TiO_2_ film exhibits an absorbance at wavelengths higher than 400 nm, which corresponds to a decrease in the band gap energy. The increase in the absorbance observed for successive deposition cycles confirms the growth of the CdS particles. No significant increase in the absorbance was observed after 15 deposition cycles.

The band gap of the films was determined by extrapolating the fitting line of the onset light absorption to zero. We have assumed that the sensitivity α·*d* (with α being the absorption coefficient and *d* being the film thickness) should be of the order of unity or *d* ≈ 1/α and that the scattering was negligible. The band gap of the TiO_2_ film is 3.08 eV, which is larger than that of bulk CdS (*E*_g_ = 2.4 eV) [1]. Increasing the number of deposition cycles leads to the onset absorption of the films being red-shifted from 333 to 518 nm, indicating a decrease of the band gap energy. The band gap decreases gradually and reaches 2.46 eV for 7×CdS/TiO_2_ and further decreases to 2.39 eV for 15×CdS/TiO_2_, which is close to the band gap of bulk CdS. The band gap of CdS decreases with the number of deposition cycles used to grow CdS on TiO_2_. This result confirms that CdS particles prepared by successive deposition cycles do possess a quantum confinement effect.

### XPS analysis of QDs-CdS/TiO_2_ films

#### Elemental analysis

**Figure 5:**
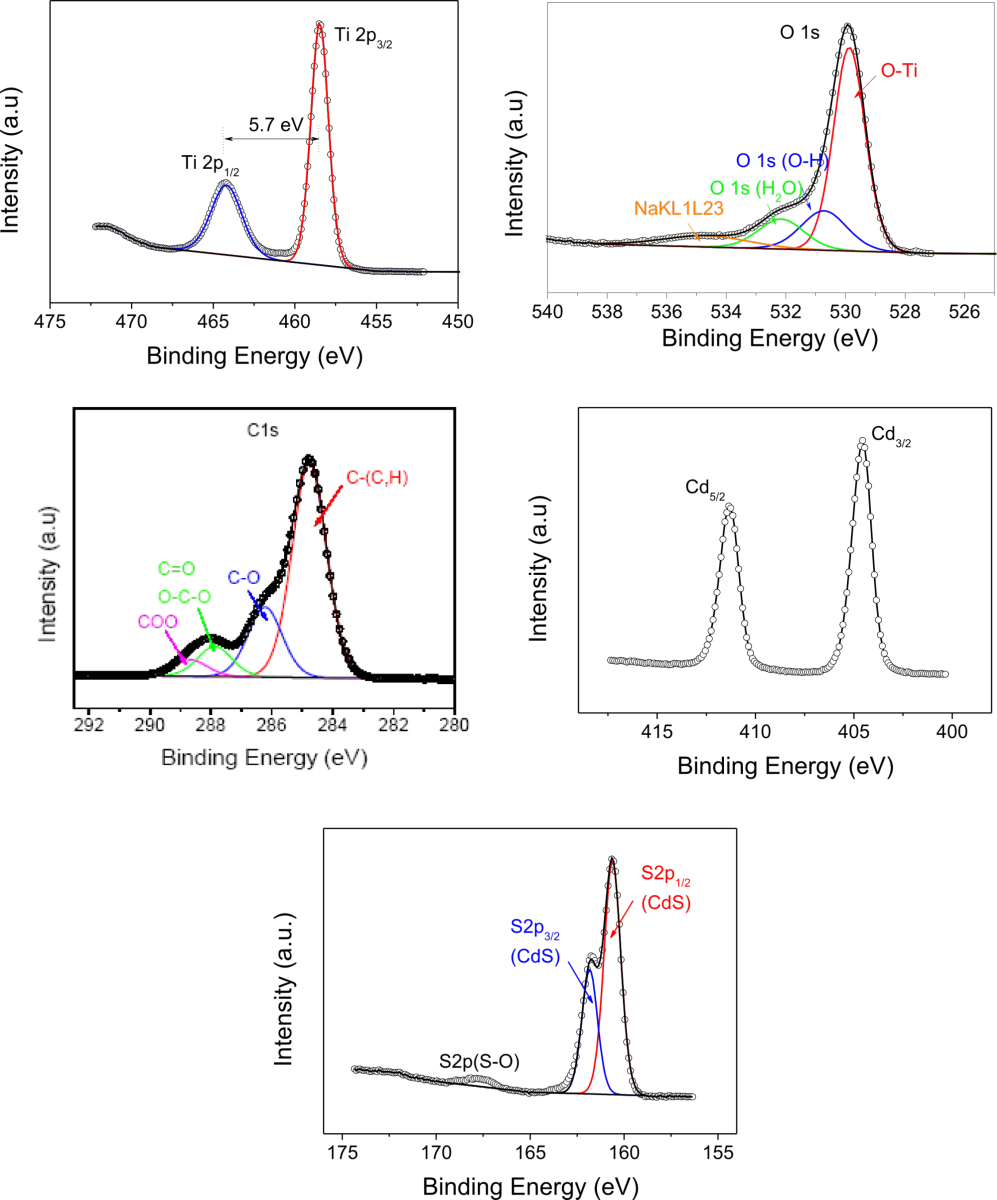
XPS analysis. Spectra of Ti 2p, O 1s, C 1s, Cd 3d and S 2p, and core peaks for 15×CdS/TiO2 sample.

The analysis was carried out on pure TiO_2_ and QDs-CdS/TiO_2_ samples. The XPS spectra of the principal elements are shown in Figure 5. The spin–orbit components (2p_3/2_ and 2p_1/2_) of the Ti 2p peak were well deconvoluted into two curves at 458.5 and 464.2 eV. The measured separation between the Ti 2p_3/2_ and Ti 2p_1/2_ peaks was 5.7 eV, which is consistent with the binding energy separation observed for stoichiometric TiO_2_ [16]. The O 1s peak was deconvoluted into three peaks at 529.8, 530.7 and 532.2 eV for all samples. These can be assigned to oxygen in the O−Ti bonds and O−H bonds of the hydroxy groups and in O−C. The deconvolution of C 1s peak results in four peaks. The one centered at 284.8 and attributed to hydrocarbon is related to the residual carbon coming from the decomposition of the titanium(IV) tetraethoxide precursor and some surface pollution during the XPS analysis. The other peaks are attributed to oxidized forms of carbons, which are usually detected (286.2 eV (C–O); 287.8 eV (C=O, O–C–O) and 288.6 eV (COO) [18]. The Cd 3d_5/2_ and Cd 3d_3/2_ were found at 411.3 and 404.6 eV respectively for QDs−CdS/TiO_2_ and were attributed to Cd^2+^ in CdS [19]. The difference between the binding energies of Cd 3d_5/2_ and Cd 3d_3/2_ is 6.7 eV, which corresponds to the presence of the oxidation state +2 of Cd 3d at the surface [20]. The S 2p_3/3_ peak (Figure 5) was found at 161.8 eV and is attributable to S^2−^ in CdS [21]. The presence of other oxidation states is shown by the peak observed at 167.5 eV, which is due to the presence of sulfate at the surface. The molar concentration of these oxidized states does not exceed 0.6%. Furthermore, no significant variation of the molar concentration of the oxidized states was observed after each step of the deposition cycles. The survey of Cl 2p and N 1s showed only the traces of nitrogen and small quantities of chlorine ions, the molar concentrations of which vary from 1.8 to 2.3% depending on the deposition cycle.

#### Determination of the QDs-CdS particle size

X-ray photoelectron spectroscopy is usually used to determine the chemical composition of the prepared samples and the valence states of the various species present. In this study we used XPS to determine the particle size of the CdS nanocrystals that were deposited on the TiO_2_ films. In the literature, there is evidence that the use of XPS signals could be a useful tool for size measurements of metallic particles [22]. The sizes of nanoparticles can be estimated from the XPS elemental intensity ratios by using an adequate modeling of the signal. Different XPS models could be applied for the estimation of average particle size [10,23–24]. Based on the diamond-shaped support-particles model described by Davis [25], (parameters reported in Table 1), which was used in this study, the average size of metallic nanoparticles was determined by evaluating the intensity ratio between two peaks of the analyzed sample.

**Table 1:** Binding energy and peak area ratio of Cd 3s and Cd 4s used for the Davis model.

sample	Cd 3s BE | [eV]	Cd 4s BE [eV]	ratio 3s/4s	particle size [Å]

CdS1	770.7	109.0	3.288	7.0
CdS2	770.4	108.8	3.165	9.0
CdS3	770.4	108.7	2.831	16.0
CdS4	770.4	108.7	2.468	28.0
CdS5	770.3	108.6	2.378	33.0
CdS6	770.3	108.7	2.017	80.0
CdS reference	770.2	108.6	1.886	—

However, these two peaks should come from two different electronic levels sufﬁciently separated in energy. In this work the Cd 3s and Cd 4s peaks were chosen as reported in Table 1. This model assumes that the electrons leave the sample under an emission angle of 45° and is more appropriate to determine the size of very small and very big particles [10]. The main advantage in using this model is a certain independence from the physical properties of the sample, such as density, pore structure or CdS loadings. The inﬂuence of the particle shape and surface roughness could be studied by using two different peaks of the same dispersed phase the intensity ratio of which is given in Equation 1:

[1]
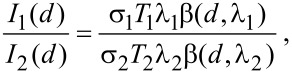


where σ is the photoionization cross section, *T* is an instrumental transmission function that reﬂects the basic detection efﬁciency, λ is the inelastic mean free path (IMFP) of the primary photoelectrons, and β is an attenuation factor, which is dependent on the particle shape and IMFP. The subscripts correspond to the two XPS peaks. Easily derived for different particle sizes by using the relation given by Davis (Equation 2), the attenuation factor (β) strongly depends on the particle shape. In this work the attenuation factor for spherical particles was used as shown in Equation 2, where *d* is the particle size and could be obtained by iteration [10].

[2]
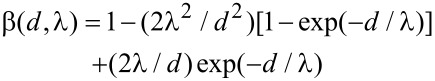


**Figure 6:**
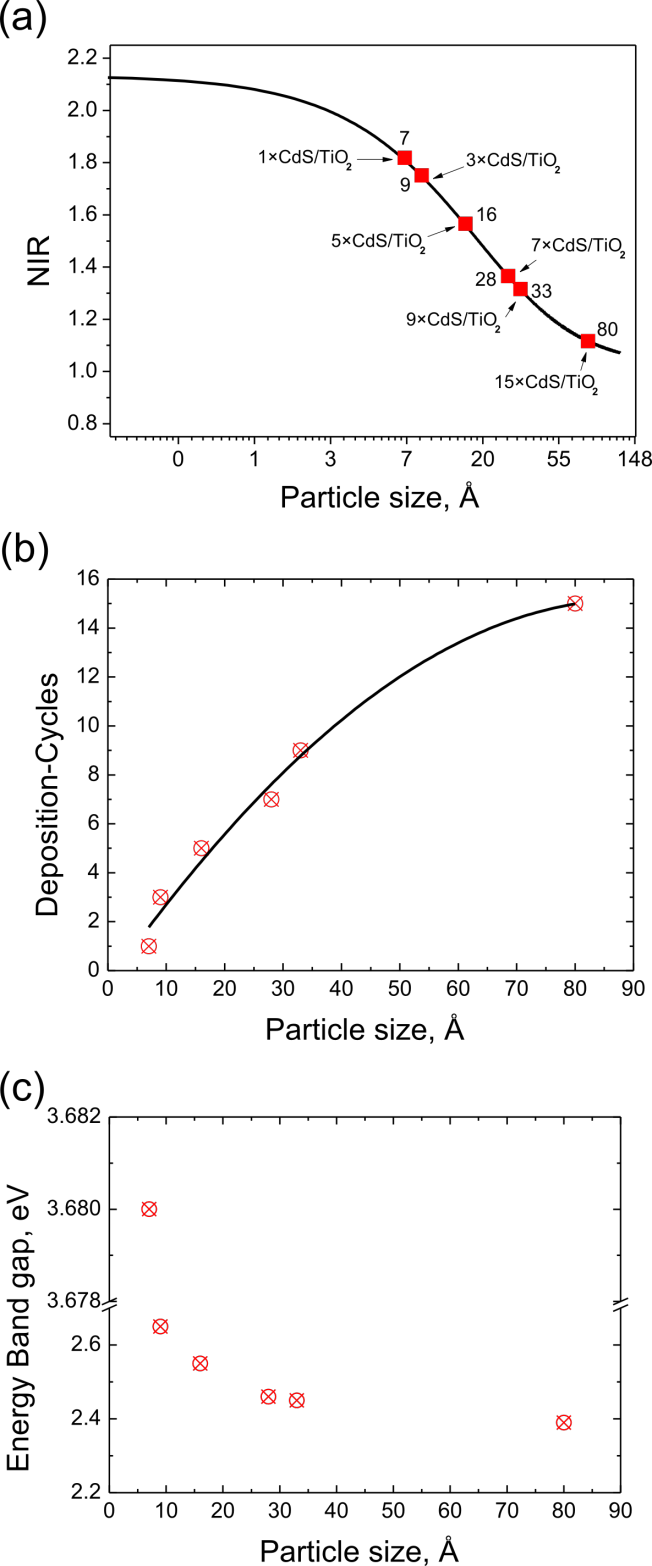
(a) CdS particle size calculated by Davis model vs NIR (normalized intensity ratio calculated from the intensity ratio of pure metal foil and studied material). Particle size evolution vs (b) number of deposition cycles and (c) the band gap energy.

The results obtained for CdS plotted by using Equation 1 and Equation 2 are shown in Figure 6a. The normalized intensity ratio (NIR) was calculated from the intensity ratio of pure CdS and the prepared samples. The main parameters are shown in Table 2. The most important parameters for applying the Davis model are the XPS peak areas and the inelastic mean free path length (λ). In our study the values of IMFP were calculated by using the Tougaard Quases-IMFP-TPP2M program [26], which is based on the algorithm proposed by Tanuma [27]. Other essential parameters such as compounds energy band gaps and the Scofield cross sections were taken from [28] and [29] respectively.

**Table 2:** XPS Parameters used in the Davis model.

	IMFP [nm]		Scofield cross section [eV]	
	λ_3s_	λ_4s_	Cd 3s	Cd 4s

Cd	0.959	2.047	3.040	0.692
CdS	1.556	2.557	—	—

The very small CdS particles were observed for the 1×CdS/TiO_2_ and 3×CdS/TiO_2_ samples (smaller than 1 nm). In contrast, the 15×CdS/TiO_2_ sample (15 deposition cycles) showed the biggest particle size (8 nm). It could be concluded that the final size of the particles could be controlled by the preparation method. Indeed, as deduced from the XPS measurements, the final CdS particle size depends on the number of deposition cycles. The smallest particles were formed on 1×CdS/TiO_2_ sample after one deposition cycle, whereas the biggest particles were prepared with 15 deposition cycles. A good correlation between CdS particle size and number of deposition cycles was observed (Figure 6b). We propose that the TiO_2_ films are covered by spherical grains, the size of which increases with the number of deposition cycles, which is in concordance with UV–vis spectroscopy and AFM studies. The small particles fill the pores of the TiO_2_ layer and then cover the surface of the substrate, which leads to a homogeneous layer. In order to illustrate the quantum size effect, the relationship between the optical band gap and the average particle size of CdS made by a different number of deposition cycles is shown in Figure 6c. As deduced from the band-gap and particle-size correlation curves, the smaller the particle size, the larger the band gap. This clearly demonstrates the quantum confinement characteristics of the CdS nanoparticles. The dependence of the optical band gap on the particle size observed in this study is consistent with previously reported data [12].

## Conclusion

This article has placed emphasis on the formation of the CdS particles on TiO_2_ films and characterizes those by using different methods. We used the XPS model for the first time, to estimate the average particle sizes of CdS quantum dots. Our results confirmed the very good dependence of the CdS particle size on the number of successive deposition cycles. Moreover, a very good correlation was observed between results obtained from XPS, AFM and UV-vis. It confirms that XPS is a powerful method for the estimation of the average particle size of CdS quantum dots. We propose that the TiO_2_ films are covered by spherical CdS nanoparticles, the size of which increases proportionally to the number of deposition cycles. The small particles accumulated continuously in the pores of the TiO_2_ layer and then covered the surface of the substrate, which leads to a homogeneous layer. After each deposition cycle the particles grew following a heterogeneous formation mechanism due to ion-by-ion deposition.
